# The PiGeOn project: protocol for a longitudinal study examining psychosocial, behavioural and ethical issues and outcomes in cancer tumour genomic profiling

**DOI:** 10.1186/s12885-018-4310-0

**Published:** 2018-04-05

**Authors:** Megan Best, Ainsley J. Newson, Bettina Meiser, Ilona Juraskova, David Goldstein, Kathy Tucker, Mandy L. Ballinger, Dominique Hess, Timothy E. Schlub, Barbara Biesecker, Richard Vines, Kate Vines, David Thomas, Mary-Anne Young, Jacqueline Savard, Chris Jacobs, Phyllis Butow

**Affiliations:** 1 Psycho-oncology Co-operative Research Group (PoCoG), University of Sydney, Level 6 North, Lifehouse (C39Z), Sydney, NSW 2006 Australia; 2 Sydney Health Ethics, Sydney School of Public Health, University of Sydney, Camperdown, NSW 2006 Australia; 3 Centre for Medical Psychology and Evidence-based Decision-making (CeMPED – Psychology), University of Sydney, Camperdown, NSW 2006 Australia; 4 Prince of Wales Clinical School, UNSW Sydney, Sydney, NSW 2052 Australia; 5 Cancer Division, Garvan Institute of Medical Research, 384 Victoria St, Darlinghurst, NSW 2021 Australia; 6 Sydney School of Public Health, University of Sydney, Camperdown, NSW 2006 Australia; 7 The National Human Genome Research Institute, National Institutes of Health, 31 Center Drive, MSC 2073, Bethesda, MD 20892 USA; 8 Rare Cancers, PO Box 440, Bowral, NSW 2576 Australia; 9 Genome One, Garvan Institute of Medical Research, 384 Victoria St, Darlinghurst, NSW 2021 Australia

**Keywords:** Tumour genomic profiling, Genome sequencing, Germline sequencing, Molecular profiling, Cancer, Psychosocial factors, Health behaviors, Ethical issues

## Abstract

**Background:**

Genomic sequencing in cancer (both tumour and germline), and development of therapies targeted to tumour genetic status, hold great promise for improvement of patient outcomes. However, the imminent introduction of genomics into clinical practice calls for better understanding of how patients value, experience, and cope with this novel technology and its often complex results. Here we describe a protocol for a novel mixed-methods, prospective study (PiGeOn) that aims to examine patients’ psychosocial, cognitive, affective and behavioural responses to tumour genomic profiling and to integrate a parallel critical ethical analysis of returning results.

**Methods:**

This is a cohort sub-study of a parent tumour genomic profiling programme enrolling patients with advanced cancer. One thousand patients will be recruited for the parent study in Sydney, Australia from 2016 to 2019. They will be asked to complete surveys at baseline, three, and five months. Primary outcomes are: knowledge, preferences, attitudes and values. A purposively sampled subset of patients will be asked to participate in three semi-structured interviews (at each time point) to provide deeper data interpretation. Relevant ethical themes will be critically analysed to iteratively develop or refine normative ethical concepts or frameworks currently used in the return of genetic information.

**Discussion:**

This will be the first Australian study to collect longitudinal data on cancer patients’ experience of tumour genomic profiling. Findings will be used to inform ongoing ethical debates on issues such as how to effectively obtain informed consent for genomic profiling return results, distinguish between research and clinical practice and manage patient expectations. The combination of quantitative and qualitative methods will provide comprehensive and critical data on how patients cope with ‘actionable’ and ‘non-actionable’ results. This information is needed to ensure that when tumour genomic profiling becomes part of routine clinical care, ethical considerations are embedded, and patients are adequately prepared and supported during and after receiving results.

**Trial registration:**

Not required for this sub-study, parent trial registration ACTRN12616000908437.

## Background

Despite treatment advances in recent years, survival rates in some cancers have only minimally improved. [1] As such, there is a significant need for new approaches to increase patient survival and reduce morbidity. Tumour genomic profiling (TGP) and personalized treatment offer new opportunities for improvement in cancer outcomes. It is anticipated that TGP will increasingly allow identification of cancer patients who will benefit from targeted drugs and immunotherapy approaches, in contrast to current ‘scatter-gun’ approaches of chemotherapy and radiotherapy. [2]

There are generally two types of genomic sequencing in the cancer context: TGP and germline genomic sequencing (GGS). TGP involves laboratory analysis of somatic tissue to identify somatic driver mutations in tumours, whereas GGS involves examination of the entire genome through a blood sample seeking germline variants associated with cancer predisposition syndromes. This study focuses on TGP. A second study examining the psychosocial, behavioural and ethical issues and outcomes of GGS on cancer probands and their blood relatives is also underway and its protocol will be published separately. The two sub-studies together represent The P (psychosocial) i(n) Ge (genomics) On (oncology) PiGeOn project.

TGP involves panel or whole genome testing of DNA derived from tumours. This may identify gene variants that can inform choice of treatments targeting the specific gene variants present in the tumour. TGP can identify somatic variants that: i) affect treatment (clinically actionable); ii) do not affect treatment (non-actionable); or iii) are of uncertain significance. TGP can be followed by further confirmatory testing if it is suspected that gene variants may have a germline origin (and therefore may also be relevant to the patient’s family).

The promise of genomic medicine will only be realised if patients understand and benefit from identification of gene variants; that is, they need to understand the chances of finding actionable variants, and any potential implications of results. While uncertainty in terms of potential information available pervades TGP less than GGS due to the smaller scope of testing, there remains an element of uncertainty due to the poor prognosis of most patients and the uncertainty of the results being clinically actionable, and also the chance of finding a germline variant with the subsequent implications of testing for the recipient’s blood relatives. Practitioners who obtain consent for TGP face the challenges of conveying these uncertainties to both ensure informed choice regarding testing and to mitigate any unrealistic expectations.

Further, although uncertainty may be shared among stakeholders, such as during education sessions between physician and patient, the extent of intricacy within genomic testing can lead to both parties remaining unaware of their areas of ignorance. [3] The expected future expansion of TGP to other healthcare providers and non-genetics specialists raises questions of 1) how best to approach the consent process, and 2) about disclosure of complex results when providers may have limited training and/or expertise in genetics. While patient autonomy, informed consent and shared decision-making are established standards in health care, there are no guidelines on how patients should be engaged with regard to the decision to have TGP, nor how uncertainty should be approached in this complex area. Debate is ongoing. [4]

We have little understanding of the ethical, psychosocial and behavioural implications of providing cancer patients with results from TGP, and whether it will be experienced differently to other medical tests. The published evidence has been gathered primarily from research participants who may or may not have cancer, where the potential benefits of a gene variant being present (such as access to novel therapeutic options) differ. Several North American studies [5–7] presenting hypothetical scenarios to cancer patients regarding TGP, found the majority would be interested in tumour profiling, although the participants felt they had insufficient knowledge to make an informed choice. While a majority of patients in hypothetical scenarios expressed a willingness to undergo TGP for personalized treatment, many also expressed significant concerns about potential psychological harm, cost and discrimination. Misunderstandings about TPG were also noted. [5]

Overall, very little is known about the knowledge, preferences, attitudes and values of cancer patients who have actually undergone TGP, their experience of uncertainty, their behaviour on receipt of TGP results, or the psychological effects these results may have. Only a handful of studies have explored responses in cancer patients who have actually been offered TGP. These have primarily noted patient report of information overload and misunderstanding, leading to unrealistic expectations, anxiety and uncertainty [8–10]. Patient hopes of benefit from TPG were enhanced by the promise of novel and targeted treatment but challenged by non-findings or by limited access to relevant trials. [8] To our knowledge, none of the studies of patients who have actually undergone TGP testing have reported longitudinal data.

Oncologists were among the first clinicians to incorporate TGP into routine management to guide treatment choice. Unifocal profiling goes back to estrogen receptor status testing in breast cancer and then EGFR mutation in lung cancer, with recent testing of the KRAS gene in colorectal cancer and the BRAF gene in melanoma. [11, 12] It is critical to explore how patients value and experience TGP, and cope with uncertainty, non-actionable results and incidental findings, as well as informative findings, as TGP increasingly enters routine oncological clinical practice.

This paper outlines the protocol of the first Australian study collecting longitudinal data on cancer patients’ experiences of TGP over time.

### Guiding theory

The design and measures for the study are guided by Protection Motivation Theory [13] and Differentiation and Consolidation Theory. [14] Protection Motivation Theory proposes that we protect ourselves based on the perceived severity and probability (vulnerability) of a threat, perceived efficacy of preventive behaviour, and perceived self-efficacy in performing the preventive behaviour. Differentiation and Consolidation theory states that decision-making reflects a process of gradual differentiation including: identifying options with perceived important attributes, prioritising one or two options based on highly ranked attributes and reconsidering an initial preference based on further information. This is followed by a consolidating process, which focuses on one’s values, and future possible outcomes, to favourably reinforce the chosen option and thereby prepare for potential threats, regret and doubt.

In this setting, protection motivation will be the result of perceived susceptibility to disease progression and death, and fear of cancer progression, as well as participant knowledge of, attitudes to, and value given to, TGP (as a strategy to guide more effective treatment and thus reduce the threat of disease progression). Patients who perceive TGP to be valuable will be more likely to remain satisfied with their decision to undertake TGP, regardless of results, and to act on positive results by joining appropriate clinical trials of tailored treatments. Intolerance of uncertainty may make TGP less attractive and increase the likelihood of regret, doubt and poor psychological outcomes.

## Methods/design

### Aims and hypotheses

The aim of this study in adults with advanced cancer undergoing TGP is to:

1. *Before and after receipt of results:* evaluate knowledge of TGP, preferences for genetic information, attitudes (positive and negative) and the value placed on TGP;
2. *After receipt of results:* identify behavioural, decisional and psychological outcomes from TGP along with their respective predictors; and
3. Contextualize the findings via a critical bioethics analysis.

Specific hypotheses are that:

1. At baseline (after giving informed consent to undergo TGP), advanced cancer patients will have moderate to poor knowledge about TGP (< 55% correct on knowledge scale).
2. At baseline, advanced cancer patients will have primarily positive attitudes to, and highly value, TGP.
3. Of advanced cancer patients *who receive an actionable result*, most will:
  - Pursue personalised treatment in a clinical trial setting (behavioural outcomes).
  - Cope well with results, and experience low decisional conflict and regret and high decisional satisfaction (decisional outcomes).
  - Have higher hope, less cancer-related anxiety, lower fear of cancer progression and lower general anxiety and depression than those who do not receive actionable results (psychosocial outcomes).
4. Those advanced cancer patients who have *no actionable variants identified* will:

- Struggle to cope with non-results (decisional outcomes).
- Have levels of hope, cancer-related anxiety, fear of cancer progression and general anxiety and depression similar to published results for advanced cancer patients who have not undergone TGP, within 1 month of receiving results and 5 months later (psychosocial outcomes).

5. Predictors of behavioural, decisional and psychological outcomes within 1 month of receiving results and 5 months later, will include:
  - Age, education level, ethnicity, and gender;
  - Tolerance of uncertainty and baseline knowledge; and
  - Preferences and attitudes to TGP.

Methodology in critical bioethics analysis is not hypothesis-driven and to this end there are no hypotheses relating to this part of the analysis.

### Parent study

The Genomic Cancer Medicine Program includes a large cohort study funded by the NSW Ministry of Health, Australia, the Molecular Screening and Therapeutics (MoST) Program. MoST is recruiting 1000 adult patients with pathologically confirmed advanced or metastatic solid cancer with a particular focus on rare or neglected cancers. Participants undergo TGP, and receive results at approximately 11 weeks. Their treating oncologist is informed of results and discusses results and treatment options with the patient. Possible treatment options identified through TGP include a clinical trial offered as part of the MoST program, another clinical trial available in Australia, or off-label treatment through the treating clinician. This cohort offers the opportunity to explore short- and long-term impacts on behaviour and psychological morbidity of receiving TGP results. The PiGeOn project constitutes a psycho-social/ethical sub-study in this cohort, employing mixed methods to gather patient-reported outcomes and qualitative data.

### Research design

This is a mixed method, prospective, cohort sub-study of a TGP programme enrolling patients with advanced cancer.

### Setting

Participants will be recruited by the parent programme, from incident and prevalent cases at three large tertiary oncology units in Sydney hospitals. All suitable participants will provide informed consent using established procedures for genetic research, validated by large genetic studies. [13, 14] The scope of this consent includes TGP as well as completion of questionnaires and interviews. Participants are provided with actionable and non-actionable results from TGP and they can elect whether they want germline genetic results confirmed by Sanger sequencing and returned. Patients for whom germline variants are found will have their decision to be informed confirmed, then directed to a genetic counsellor for further information.

### Participants

The target population includes patients with pathologically confirmed advanced or metastatic solid cancer of any histologic type. Patients with rare or neglected cancers (defined as an incidence of less than 6/100,000 of the general population and cancers of unknown primary site) will be given priority.

*Inclusion criteria* include: age 18 years or older; pathologically confirmed advanced and/or metastatic solid cancer or an earlier diagnosis of a poor prognosis cancer; sufficient and accessible tissue for TGP; failed all standard anticancer therapy or receiving last line of standard therapy at time or enrolment; Eastern Cooperative Oncology Group Performance Status Measure (ECOG) performance status 0, 1 or 2; willing and able to comply with all study requirements, including timing and/or nature of required assessments; and written informed consent.

*Exclusion criteria* include: suitability for standard therapy, if the patient has not been previously treated; specific co-morbidities or conditions which may contraindicate participation or compromise assessment of key outcomes; history of another malignancy within 2 years prior to registration: pregnancy, lactation or inadequate contraception.

### Procedure for PiGeOn

**Fig. 1:**
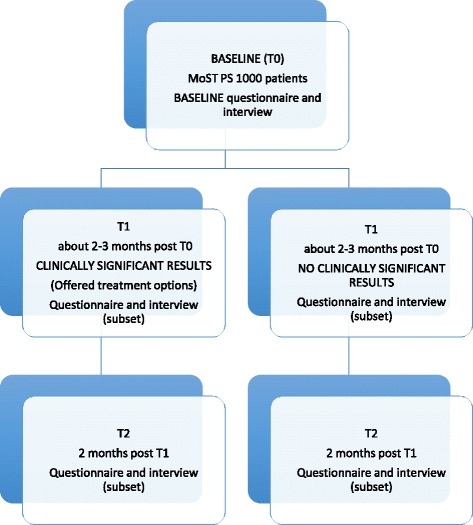
Assessment schedule. MoST PS: MoST Psychosocial (PiGeOn) sub-study

Patients will provide consent to MoST staff for the psychosocial (PiGeOn) sub-study at the same time as they give consent to the parent TPG program. Thus at baseline they will have already considered and given consent to TGP, but not yet had TGP. Participants will be asked to complete a questionnaire (hard copy, online or via telephone if they prefer) at baseline (T0), 1 to 4 weeks after receiving TGP results (T1, approximately 2–3 months post baseline), and at 2 months (T2) after T1. These timeframes have been chosen to allow impacts of the test results to be captured in both the short-term (when a treatment decision is being made) and longer-term (while undergoing or near to completion of targeted therapy, if adopted). A small subset (around 20–40 participants) will be invited to participate in semi-structured interviews at the same assessment time points to explore in more detail attitudes towards TPG and its associated psycho-social, behavioural and ethical aspects. See Fig. 1.

### Measures

The following patient-reported measures will be collected (see Table 1).

**Table 1:** Survey measures

Domain	Measures	Baseline (T0)	Follow-up 1 (T1) (within 4 weeks of return of results, ~ 2–3 months)	Follow-up 2 (T2) (2 months post T1)
Demographics				
Age, gender, marital status, education level, occupation etc	Demographic items	X		
Views and attitudes				
Value of TGP	Adapted from previous studies [15–17]	X		
Perceived benefits and drawbacks of TGP	Adapted from Jamal et al. [18]	X	X	
Result return preferences	Adapted from Tabor et al. [19]	X		
Knowledge	Study-developed	X		
Perceived importance of TGP	Adapted from Hay et al. [20]	X		
Psychological predictors				
Coping with uncertainty	Kasparian et al. [21]	X		
Self-efficacy	Adapted from Rosenberg et al. [22]	X		
Perceived susceptibility	Adapted from Kasparian et al. [21]	X	X	X
Psychological outcomes				
Coping with TGP results	Multidimensional impact of cancer risk assessment [23]		X	
Fear of cancer progression	Concerns about Recurrence Questionnaire [24]	X	X	X
Cancer -specific anxiety	Impact of events scale [25]		X	X
Anxiety and depression	Hospital anxiety and depression scale [26]		X	X
Hope	Herth Hope Index [27]		X	X
Decisional outcomes				
Decisional conflict	Decisional conflict scale [28]		X	
Decisional regret regarding personalised treatment	Decision regret scale [29]			X
Decisional satisfaction	Satisfaction with Decision Scale [30]	X	X	

TGP, Tumour genomic profiling

### Demographic data

Age, gender, marital status, education level, occupation, language spoken at home and history of genetic screening collected by patient report at baseline. Sociodemographic status will be derived from National Census data based on location of residence.

### Disease details

Available from the MoST database, including family history, primary site, detailed staging, ECOG performance status, treatment and co-morbidities.

The following validated (some adapted) outcome measures will be administered at baseline only:

### Perceived importance of TGP

This 2-item measure adapted from Hay et al. [15] assesses perceived importance of genetic information to the participant, using a Likert scale. Specifically, the questions pertain to importance of learning about how genes affect the chance of responding to particular cancer treatment, and how lifestyle affects the chance of living longer with cancer. High scores indicate greater importance.

### Knowledge

A 9-item, multiple choice, study-developed questionnaire assessing knowledge of the purpose of TGP, likely frequency of informative results, cancers in which informative results are more likely to be found, availability of tailored treatment options, and source of genetic knowledge. Scores are summed, with high scores indicating greater knowledge.

### Self-efficacy

Four items adapted from Rosenberg et al., [16] assessing perceived ability to cope if actionable, non-actionable, or germline results are found. High scores indicate greater perceived ability to cope.

### Value of TGP

A hypothetical time trade-off scenario based on those used in three previous studies. [17–19] Six items assess how the likelihood of finding an informative result impacts willingness to have TGP, and the amount the participant would be willing pay for TGP (from $0 to $10,000) if TGP found an informative result in 1, 20, or 40 people out of 100.

### Preferences for being informed of results

Four Likert-scale items adapted from Tabor et al., [20] assessing desire for results informing: treatment, prognosis, and family risk of cancer (yes / no / maybe / don’t know).

### Coping with uncertainty

Eight Likert-scale items from Kasparian et al., [21] measuring reaction to uncertainty, ambiguity and the future. High scores indicate greater intolerance.

Note that to reduce participant burden at baseline, measures of psychological morbidity (anxiety, depression, hope) were not included at T0.

The following outcome measures will be administered at the first follow-up only:

### Coping with genetic test results

The 25-item Multidimensional Impact of Cancer Risk Assessment [22] assessing impact of result disclosure after genetic testing. High scores indicate greater distress.

### Decisional conflict regarding tailored treatment

Ten items from the Decisional Conflict Scale [23] comprises 5 subscales measuring: 1) uncertainty, 2) feelings of being uninformed, 3) clarity of values, 4) sense of being unsupported in decision-making, and 5) evaluation of the quality of the decision. High scores indicate lower decisional conflict.

The following outcome measures will be administered at the first and second follow-ups:

### Cancer-related anxiety

The 15-item Impact of Events Scale [24] assesses cancer-related anxiety, in two subscales, intrusive thinking and avoidance. High scores indicate greater cancer-related anxiety.

### Anxiety and depression

The 14-item Hospital Anxiety and Depression Scale [25] comprises two 7-item sub-scales measuring anxiety and depression. High scores indicate greater morbidity.

### Hope

The 12-item Herth Hope Index [26] measures hope and sense of meaning, with 3 subscales: temporality and future, positive readiness and expectancy, and inter-connectedness. High scores indicate greater hope.

The following outcome measure will be administered at the second follow-up:

### Decisional regret

The 5-item Decisional Regret Scale [27] measures health care decision regret about the decision to have TGP. High scores indicate greater regret.

The following outcome measures will be administered at baseline and first follow-up:

### Perceived benefits and drawbacks of TGP

Two open ended questions adapted from an earlier study [28] assessing perceived specific benefits and drawbacks of TGP.

### Satisfaction with decision to have TGP

The 6-item Satisfaction with Decision scale [29] measures satisfaction with decision to have TGP. Items are rated on a Likert scale. High scores indicate greater satisfaction.

The following outcome measures will be administered at baseline and all follow-ups:

### Fear of cancer progression

Three items from the Concerns about Recurrence Questionnaire [30], adapted to measure fear of cancer progression. High scores indicate greater fear.

### Perceived susceptibility

Participants indicate perceived likelihood of having a gene fault that increases risk of cancer progression on a visual analogue scale (0–100%) adapted from Kasparian et al. [21]

### Qualitative interviews

A subset of participants will be invited to participate in three semi-structured interviews (at baseline and each follow-up). Recruitment will continue until saturation is reached. [31] As the majority of this sample population is expected to have non-actionable results, additional participants, purposively sampled to include those who have received actionable or incidental findings, will be recruited at follow-up 1 and re-interviewed at follow-up 2. Similarly, purposive sampling will be used to ensure that both patients who elect to receive, and not receive, germline results (if available) will be included. Also, the opportunity to contrast TGP with the experience of having previously undergone testing for a single variant to personalize treatment will be taken where possible. Interviews will explore views on who should be offered TGP, attitudes to disclosure of results, perceived benefits and challenges of TGP, ethical aspects of TPG testing and experiences of receiving informative and uninformative results.

### Sample size

The MoST study plans to recruit 1000 patients over three years. Thus, with a conservative retention and survival estimate of 75%, this is reduced to at fewest 469 patients. With this sample size estimate, assuming 15% of patients receive an actionable result and using a significance level of 0.05, this project: has 90% power to detect a mean change of 5.8 and 2.6 points on the Impact of Events scale for patients with actionable and non-actionable results respectively (paired t-test, SD = 15) [32] and a mean difference of 4.5 points between patients with actionable and non-actionable results; Multiple regression with at most 24 explanatory variables (including dummy variables) has 90% power to detect significant categorical variables with 5 categories (largest possible categorical variable) when that predictor explains greater than 3.6% of the residual variance, or to detect a continuous variable when it explains more than 2.3% of the residual variance. For patients with actionable results only (at fewest 70), at 80% power the minimum variance explained for continuous variables would be 12% of the residual variance, and 15% for binary predictors. For logistic regression when outcomes are on average equally likely the power to detect an odds ratio of 1.4 is 95% or 80% when the other covariates explain zero, or 37% of the outcome variance respectively. For only those with actionable results, there is 80% power to detect an odds ratio of 2.14 or greater if the other outcomes explain zero outcome variance.

### Qualitative data analysis

Interviews will be recorded and transcribed verbatim and manually coded line-by-line. Transcripts will be subjected to Framework Analysis, [33] allowing comparisons within and between transcripts. QSR N-Vivo 11 will be used to manage the dataset. A multi-disciplinary team will be involved in analysis to ensure reflexivity. The resulting data will be cross-referenced with quantitative data to provide greater richness to the data-set. This data will also be critically interrogated as part of the ethical analysis. [34]

### Ethical analysis

Ethical concepts and analysis will be relevant throughout this project, and will be iteratively and critically reflected upon as the study progresses. Quantitative survey items allowing free-text responses regarding benefits and drawbacks of TGP will allow the study team to see whether any responses assist in identifying relevant ethical themes; such as how participants view autonomous decision-making or cost considerations. Data from qualitative interviews will be critically compared with bioethics literature regarding concepts such as family communication, duties to disclose, what constitutes autonomous decision-making, approaching uncertainty in TGP, the distinction between research and clinical practice, obligations at the conclusion of a research project and determining when it is appropriate to offer testing; and to whom. The result will be a series of normative positions, defensible using ethical reasoning and informed by empirical data.

### Quantitative data analysis

Mean differences in outcomes will be compared using t-test (continuous) or chi-squared tests (dichotomous). Non-parametric tests will also be used where appropriate. Temporal changes in scales will be investigated by calculating the difference between time-points. Multiple (continuous outcomes) or logistic (dichotomous outcomes) regression will be used to adjust for the effect of confounders and identify predictors of outcome. Linear mixed models and logistic mixed models will be carried out in R: A language and environment for statistical computing using package nlme for the linear mixed model and lme4 for the logistic mixed model. Assumptions of normality of residuals and homogeneity of variance will be checked visually though diagnostic residual plots. Multivariable models will be constructed with the inclusion of all potential confounders, and those predictors that show weak evidence for an association with the outcome in univariate analysis. Backwards elimination followed by forwards addition will be used to select predicting variables in the final model. Known and identified confounders will be included regardless of their statistical significance. Collinear independent variables will be identified and removed.

## Discussion

The PiGeOn study advances the field by identifying the knowledge, values, attitudes and coping strategies of patients with regards to TGP, determining how these, and other factors, predict subsequent cancer-related behaviour and psychosocial outcomes, and aligning these with current ethical norms. It will be the first Australian study to collect longitudinal data on cancer patients’ experience of TGP over time.

The trial experience of the first 100 patients revealed the unexpected finding that a significant proportion of the cohort was eligible for both this sub-study, and the twin psychosocial sub-study, which involved participants undergoing GGS. This group will be treated as a separate cohort, as they will be facing the combined challenges of uncertainty related to prognosis/treatment related outcomes of the MoST component, as well as the germline implications for identifying increased cancer risk for blood relatives. Previous research using hypothetical scenarios unsurprisingly suggests that patients with cancer have greater concerns about GGS than TGP. [5] This study will provide the opportunity to explore attitudes of patients who have elected to undergo both.

Methodological issues considered in the development of this study included the timing of the psychosocial assessments, including questionnaires. Timing was crucial in order to capture participant experiences and this was modified early based on data collected in the initial phase of the programme. Given it is expected that 5–10% of the cohort will have actionable somatic mutations, sampling for the qualitative component will be manipulated between baseline and follow-up 1 to allow for exploration of the reaction to both return and non-return of actionable results. The follow-up 1 cohort will be reassessed qualitatively at the subsequent follow-ups. It is recognized that there may be challenges in maintaining the follow-up of patients not receiving actionable results, although cancer populations are known to be highly motivated to contribute to research. [35] It will be important to capture the experience of having hopes of finding a novel cure raised in 100% of poor prognosis patients only to have them dashed in the at least 90% in whom nothing is found. [36]

Given the information needs and preferences are expected to vary, the qualitative and ethical components have been included to capture and critically reflect on the complexity of and nuances within patient expectations, their understanding of the implications of TGP, the likelihood of receiving an actionable somatic result and their attitudes towards sharing results with potential familial repercussions even if they did not receive such a result. Qualitative research has value in understanding these processes. [37]

Genomic sequencing (tumour and germline) is likely to become pervasive across healthcare, and to influence cancer diagnosis, treatment and prognostication. It gives rise to testing methods that are both diagnostic and predictive on a scale not previously seen in healthcare. The PiGeOn study results will inform ongoing ethical debate on issues relevant to the large-scale introduction of genomic testing into the clinical space, such as guidelines for truly obtaining informed consent for genomic testing as well as assenting to unknown future research. In addition, although somatic testing rarely identifies germline mutations, relevant to family members, it has already been reported in our study and others. [38] The PiGeOn Project will also provide critical outcome data concerning the psychosocial impact of receiving genomic results on patients. These data are needed to ensure that when genomic testing is introduced into routine clinical care, ethical concepts are well embedded, and patients are adequately prepared and supported during and after the testing process.

Given the dearth of information on participant responses to actual TGP for cancer, this study is an important step in filling the gap in knowledge about how best to introduce this form of genomic testing into clinical practice.

## Abbreviations

BRAF: proto-oncogene B-Raf
DNA: Deoxyribonucleic acid
GGS: Germline genomic sequencing
KRAS: Kirsten ras oncogene
MoST: The Molecular Screening and Therapeutics Program
SD: Standard deviation
TGP: Tumour genomic profiling
